# LITES-Based Sensitive CO_2_ Detection Using 2 μm Diode Laser and Self-Designed 9.5 kHz Quartz Tuning Fork

**DOI:** 10.3390/s25072099

**Published:** 2025-03-27

**Authors:** Junjie Mu, Jinfeng Hou, Shaoqi Qiu, Shunda Qiao, Ying He, Yufei Ma

**Affiliations:** 1 National Key Laboratory of Laser Spatial Information, Harbin Institute of Technology, Harbin 150001, China; junjiemu@126.com (J.M.); 23s121081@stu.hit.edu.cn (J.H.); yinghe@hit.edu.cn (Y.H.); 2 Zhengzhou Research Institute, Harbin Institute of Technology, Zhengzhou 450008, China; 3 School of Physics, Harbin Institute of Technology, Harbin 150001, China; 2022110064@stu.hit.edu.cn

**Keywords:** carbon dioxide (CO_2_), quartz tuning fork, light-induced thermoelastic spectroscopy (LITES), gas sensing

## Abstract

A carbon dioxide (CO_2_) sensor based on light-induced thermoelastic spectroscopy (LITES) using a 2 μm diode laser and a self-designed low-frequency trapezoidal-head QTF is reported for the first time in this invited paper. The self-designed trapezoidal-head QTF with a low resonant frequency of 9464.18 Hz and a high quality factor (*Q*) of 12,133.56 can significantly increase the accumulation time and signal level of the CO_2_-LITES sensor. A continuous-wave (CW) distributed-feedback (DFB) diode laser is used as the light source, and the strongest absorption line of CO_2_ located at 2004.01 nm is chosen. A comparison between the standard commercial QTF with the resonant frequency of 32.768 kHz and the self-designed trapezoidal-head QTF is performed. The experimental results show that the CO_2_-LITES sensor with the self-designed trapezoidal-head QTF has an excellent linear response to CO_2_ concentration, and its minimum detection limit (MDL) can reach 46.08 ppm (parts per million). When the average time is increased to 100 s based on the Allan variance analysis, the MDL of the sensor can be improved to 3.59 ppm. Compared with the 16.85 ppm of the CO_2_-LITES sensor with the commercial QTF, the performance is improved by 4.7 times, demonstrating the superiority of the self-designed trapezoidal-head QTF.

## 1. Introduction

Carbon dioxide (CO_2_) is a colorless and odorless gas [1]. As one of the major greenhouse gases, it has a profound impact on the climate system [2]. Industrial emissions [3] and the combustion of fossil fuels [4] are exacerbating the increase in the concentration of CO_2_ in the atmosphere. With the growth of the CO_2_ concentration, various environmental problems, such as global warming, will occur. In the medical field, detecting the concentration of CO_2_ in exhaled gas is also a method to help diagnose some respiratory diseases [5]. In agriculture, the impact of CO_2_ concentration on the nutritional quality of crops cannot be ignored [6]. Therefore, the detection of CO_2_ concentration is of great importance, and the development of highly sensitive CO_2_ sensors is urgent.

There are many types of gas sensors, such as mechanical, chemical, thermal, and optical [7,8,9,10,11,12,13,14,15]. Among them, laser absorption spectroscopy (LAS), an optical detection method with numerous advantages [16,17,18,19,20,21,22,23,24], stands out. This method has many advantages, including fast response speed, good stability, high sensitivity, and excellent selectivity. In 2002, as an LAS technique, quartz-enhanced photoacoustic spectroscopy (QEPAS) was initially put forward [25]. This method uses a quartz tuning fork (QTF) to replace the microphone used as an acoustic transducer in traditional photoacoustic spectroscopy (PAS). The high quality (*Q*) value and narrow bandwidth of the QTF greatly improve the anti-interference ability and frequency selectivity of the QEPAS technology [26,27]. The small size of the QTF also reduces the size of the detection element. However, the QEPAS method requires the QTF to be placed directly in the gas to be measured, meaning that contact measurements can be performed. When measuring high-temperature or corrosive gases, for example, in automobile engines and when detecting corrosive gases such as hydrogen sulfide, the QTF is prone to rapid corrosion and damage, significantly reducing its detection performance [28]. In 2018, the light-induced thermoelastic spectroscopy (LITES) technique was proposed by Ma et al. [29]. In this technology, the QTF is no longer placed in the target gas. Instead, the light beam passing through the gas is directly irradiated onto the stem of the QTF. Due to the laser-induced thermoelastic effect, the QTF will generate mechanical vibration [30]. When the modulation frequency of the laser is the same as the resonant frequency of the QTF, the mechanical vibration of the QTF will be enhanced. The mechanical vibration is converted into an electrical signal through the piezoelectric effect of the QTF. The electrical signal is extracted by the electrodes on the surface of the QTF and then the transmitted electrical signal is demodulated to deduce the concentration of the gas to be measured [31,32]. The LITES technology has successfully solved the problems faced by QEPAS, enabling the QTF to be outside the measured environment and achieving non-contact measurement. To date, various gas detection methods based on LITES technology have been reported [33,34,35,36,37,38,39,40].

In LITES technology, the QTF plays a decisive role in the performance of the system [41]. So far, the most commonly used QTF is the commercial QTF, which has the advantages of low price and stable performance, with a resonant frequency of 32.768 KHz. However, the magnitude of the signal level in the LITES sensor is related to the laser energy absorbed by the QTF [42]. The lower the resonant frequency of the QTF, the longer the energy accumulation time, resulting in a higher signal level. However, the resonant frequency should not be too low, as an excessively low frequency increases sensitivity to environmental noise and slows down the response speed [43,44,45,46]. The resonant frequency is determined by the size of the tuning fork prongs [47]. By selecting different prong shapes and sizes, a low-frequency QTF can be obtained, which can then be used as the detection unit in LITES technology to enhance the signal level.

In this paper, a highly sensitive CO_2_-LITES sensor using a 2.004 µm continuous-wave (CW) distributed-feedback (DFB) diode laser and a self-designed low-frequency trapezoidal-head QTF is reported for the first time. The QTF with a resonant frequency of 9464.18 Hz can significantly increase the accumulation time and signal level. A comparison between the commercial QTF and the trapezoidal-head QTF is performed to show the advantages of the self-designed trapezoidal-head QTF. The long-term stability of the system is analyzed using Allan deviation analysis.

## 2. Experimental Setup

### 2.1. Selection of the CO_2_ Absorption Line

**Figure 1:**
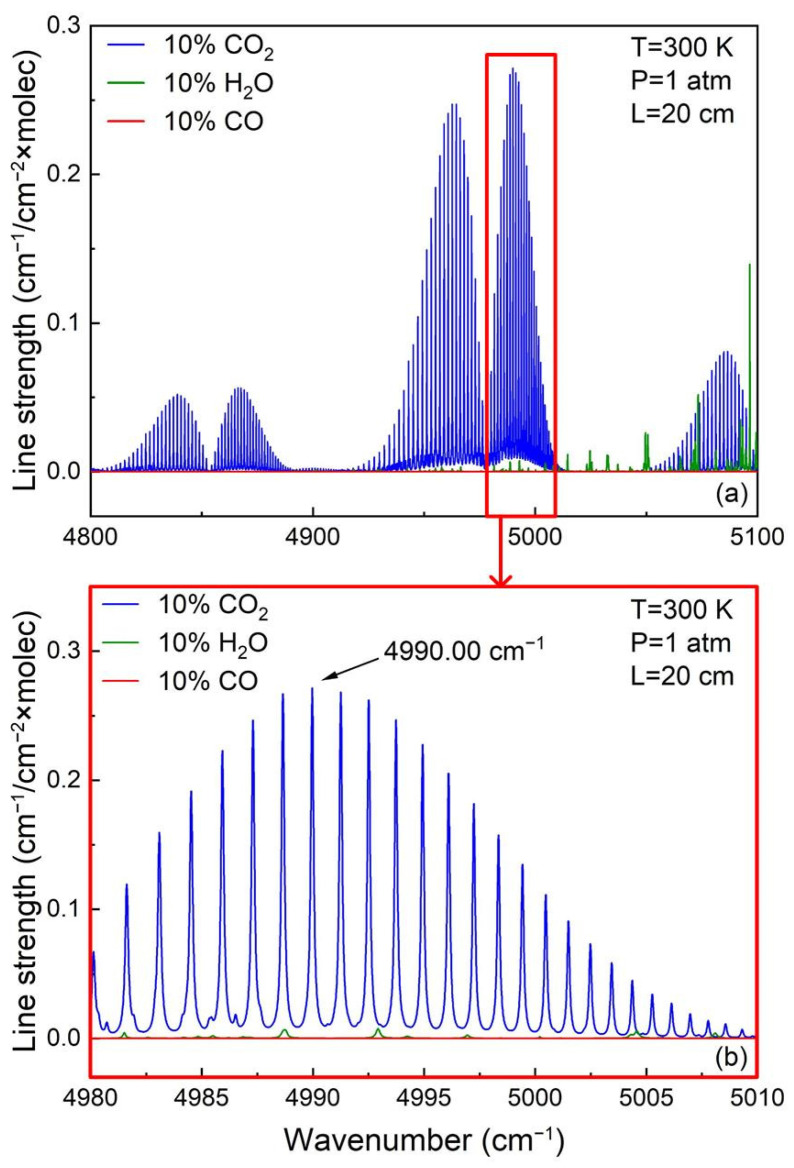
CO_2_, CO, and H_2_O absorption lines simulation based on the HITRAN 2023 database. (**a**) CO_2_, CO, and H_2_O absorption line intensity in the range of 4800–5100 cm^−1^; (**b**) CO_2_, CO, and H_2_O absorption line near 4990 cm^−1^.

In the LITES sensor system, selecting the appropriate gas absorption lines is critical. The following three principles should be obeyed: (1) the selected gas absorption line should have a relatively strong absorption coefficient; (2) the availability of obtaining a laser that matches the frequency of the selected gas absorption spectral line; (3) the selected gas absorption spectral line should not interfere with the non-target gases. Based on the HITRAN 2023 database, as can be seen in Figure 1, under the conditions of a temperature of 300 K, an absorption length of 20 cm, and a CO_2_ gas concentration of 10%, the intensity of the gas absorption spectral lines of CO_2_ in the range of 4800–5100 cm^−1^ is significantly stronger than that of water vapor (H_2_O) and carbon monoxide gas (CO), and the absorption spectral line at 4990 cm^−1^ has the highest intensity in this region. Therefore, a continuous-wave distributed-feedback (CW-DFB) diode laser with a wavelength of 2004.01 nm was selected as the laser source in the experiments.

**Figure 2:**
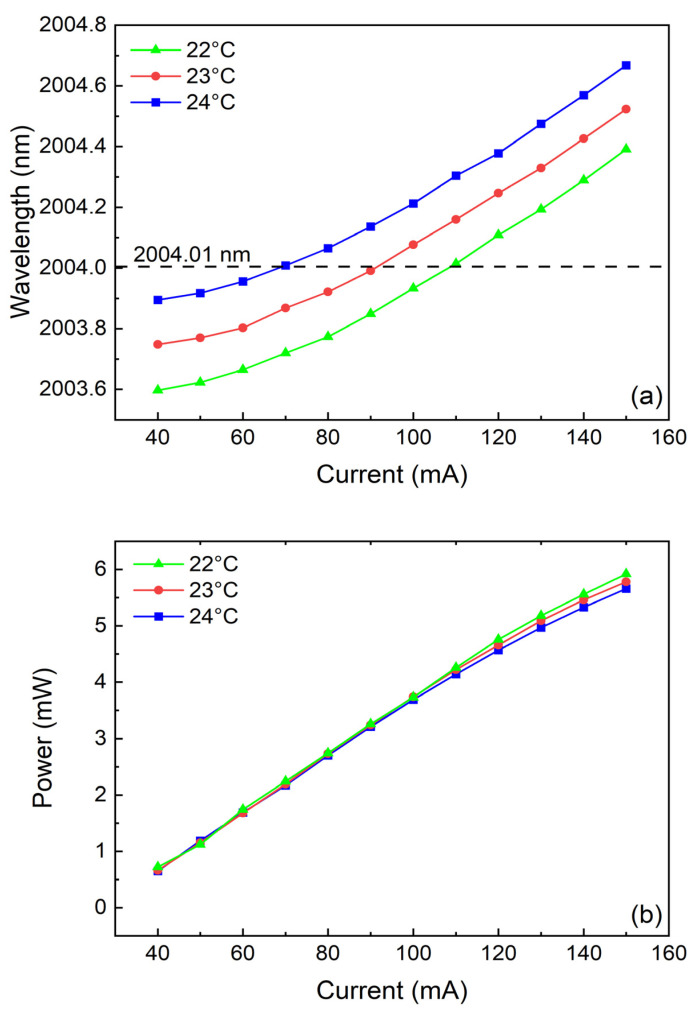
Output characteristic of the used 2 μm diode laser: (**a**) output laser wavelength varying with injection current and temperature; (**b**) output power varying with injection current and temperature.

The selection of the laser light source has a significant impact on the performance of the system. In photoacoustic spectroscopy technology, the excitation sources that have been utilized include CW-DFB diode lasers, Q-switched fiber lasers, distributed feedback quantum cascade lasers (QCL), etc. Among them, Q-switched fiber lasers are prone to environmental interference, sensitive to external temperature and vibration, and fiber fusion and coupling are highly difficult. DFB-QCLs usually require additional refrigeration equipment and are expensive. Therefore, a CW-DFB diode laser is selected as the excitation source for this system. This system employs a CW-DFB diode laser with a central emission wavelength of 2004.01 nm. The output characteristic diagram of this laser is shown in Figure 2. As the injection current increases, the output wavelength of the laser also increases. When the temperature is set at 23 °C and the injection current is 88.2 mA, the output wavelength of the laser is 2004.01 nm, which exactly matches the absorption line of the target CO_2_.

### 2.2. The Characteristics of Self-Designed Trapezoidal-Head QTF

In LITES sensors, the detection limit of the system can be significantly improved by rationally designing parameters such as the shape of QTF. In order to obtain a stronger electrical level signal, the energy accumulation time (*t*) of the QTF should be increased, and its relationship is as follows [48]:

(1) $$t=\frac{Q}{f_{0}}$$

where *Q* is the quality factor of the QTF, and *f*_0_ is the resonant frequency of the QTF. A longer energy accumulation time can be achieved by reducing the resonant frequency of the QTF and increasing the *Q* value. The magnitude of the resonant frequency is related to the length, spacing, thickness, etc., of the fingers of the QTF. The value of *Q* is related to the length and spacing, and their relationship is as follows:

(2) $$f_{0}=\frac{\pi D}{8\sqrt{12}L^{2}}\sqrt{\frac{E}{\rho }}n^{2}$$

(3) $$Q\propto \frac{WD}{L}$$

Among them, *L*, *D*, and *W* are the finger length, thickness, and spacing of the QTF fingers, respectively. It can be seen that by increasing the finger length and reducing the thickness of the QTF, the resonant frequency of the QTF can be reduced. A trapezoidal head is added to the upper end of the QTF tines. This design raises the overall center of gravity of the QTF and amplifies its vibration, thereby increasing the amplitude of the QTF during oscillation and enhancing its electrical signal level.

**Figure 3:**
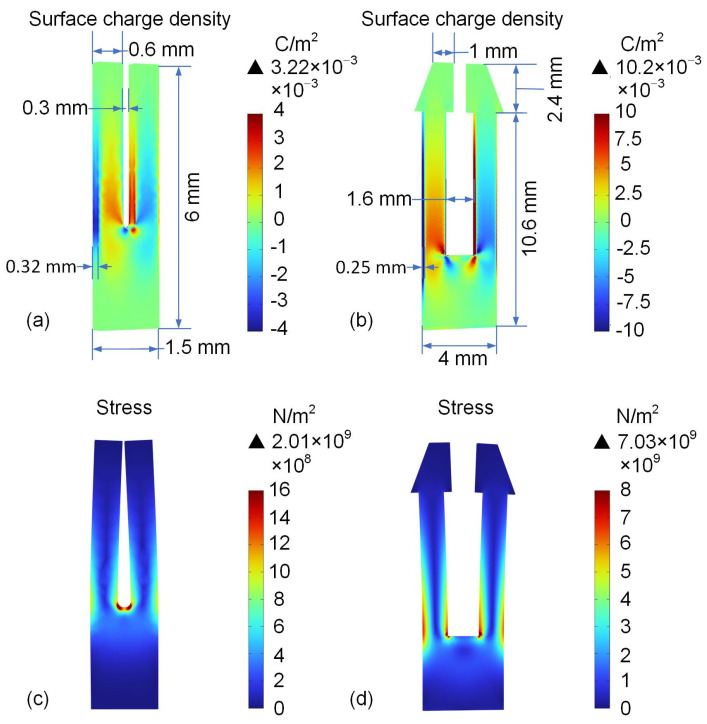
Simulation of stress and surface charge density distribution under frequency-domain excitation: (**a**) surface charge density simulation of commercial QTF; (**b**) surface charge density simulation of the self-designed trapezoidal-head QTF; (**c**) stress simulation of commercial QTF; (**d**) stress simulation of the self-designed trapezoidal-head QTF.

The COMSOL Multiphysics 6.2 simulation software is used to optimize the parameters of the QTF to reduce *f*_0_. In order to achieve the best results, several parameters that affect the performance of the QTF mentioned above are used to optimize the shape of the trapezoidal-head QTF. The dimensions of the commercial QTF and the self-designed trapezoidal-head QTF are shown in Figure 3a and Figure 3b, respectively.

First, in order to study the vibration performance of the self-designed trapezoidal-head QTF and the commercial QTF, the solid mechanics module, electrostatic module, and piezoelectric multi-physics fields in COMSOL are utilized. Through the eigenfrequency and frequency-domain studies, the vibration frequencies *f*_0_ of the commercial QTF and the trapezoidal-head QTF are obtained as 32,444 Hz and 9563.7 Hz, respectively. The maximum surface charge density and the surface charge integral of the trapezoidal-head QTF are both higher than those of the standard QTF, as shown in Figure 3a,b. The maximum surface charge density of the trapezoidal-head QTF is 3.17 times that of the commercial QTF. The surface charge integrals are 7.23 × 10^−9^ C and 2.41 × 10^−8^ C, respectively, with the former being 3.33 times higher than the latter. The trapezoidal head causes the QTF to generate greater stress during vibration, as shown in Figure 3c,d. It can be seen that the maximum stress of the trapezoidal-head QTF is 3.50 times greater than that of the commercial QTF.

**Figure 4:**
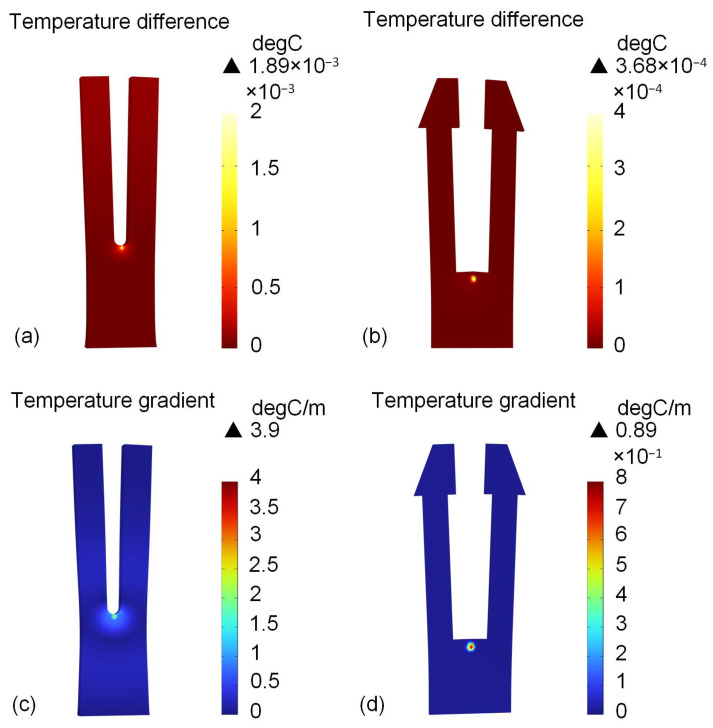
Temperature difference and temperature gradient variation in LITES simulation: (**a**) temperature difference in commercial QTF; (**b**) temperature difference in the self-designed trapezoidal-head QTF; (**c**) temperature gradient of commercial QTF; (**d**) temperature gradient of the self-designed trapezoidal-head QTF.

LITES simulations are carried out for the two types of QTFs. In LITES, the modulated laser irradiates the surface of QTF, causing the periodic thermal expansion of QTF and thus generating vibrations. Therefore, multi-physics fields such as solid mechanics, heat transfer in solids, geometric optics, and thermal expansion are selected for use. The laser power is set to 10 mW, and sinusoidal wave modulation at the corresponding resonance frequency is performed. Through transient analysis, the surface temperatures of the commercial QTF and the trapezoidal-head QTF are obtained, as shown in Figure 4a and Figure 4b, respectively. It can be seen that the temperature difference in the trapezoidal-head QTF is 5.14 times higher than that of the commercial QTF. In Figure 4c,d, the temperature gradient of the trapezoidal-head QTF is 4.38 times bigger than that of the commercial one, and the temperature gradients are 3.9 C/m and 0.89 C/m, respectively.

### 2.3. Schematic Diagram of the Experimental Setup

**Figure 5:**
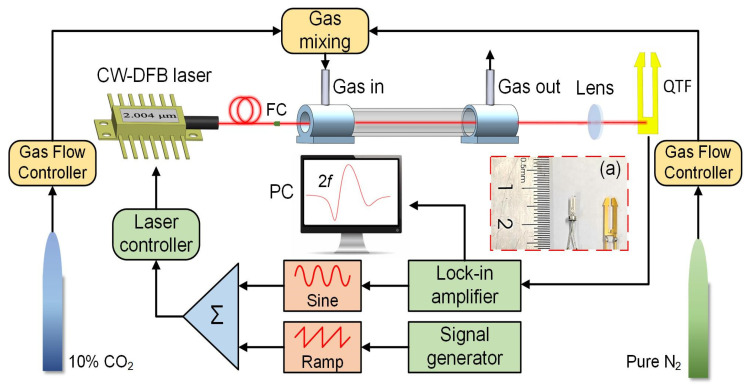
Schematic diagram of the experimental setup for the CO_2_-LITES sensor.

Figure 5 shows the experimental setup of the CO_2_-LITES sensor using a laser central emission wavelength of 2004.01 nm. After the laser is emitted from the CW-DFB laser, it is collimated by a fiber collimator (FC). The laser enters the gas cell where the light absorption occurs. Use a mass flow controller (MFC, Sevenstar, Beijing, China) to prepare CO_2_ with different concentrations. After exiting from the cell, the laser is converged by a lens to the stem of QTF to obtain the maximum thermoelastic signal. Figure 5a shows the commercial QTF and the self-designed trapezoidal-head QTF used in the experiment. The commercial QTF is relatively small in size, with the length of the fork being approximately 0.4 cm. The self-designed trapezoidal-head QTF has a low resonant frequency of 9464.18 Hz. At the same time, the unique trapezoidal-head design of the fork can amplify the amplitude of the mechanical vibration of the QTF, thereby enhancing the sensor level signal. To reduce the influence of environmental noise, this research uses wavelength modulation spectroscopy and the second harmonic (2*f*) demodulation technique. Wavelength modulation can enable the laser wavelength to accurately sweep across the absorption line. The 2*f* demodulation technology can make the signal positively correlated with the concentration, and it has a strong inhibitory effect on environmental noise and the low-frequency noise of the instrument itself. The high-frequency sine wave generated by the lock-in amplifier (Zurich Instruments, Zurich, Switzerland) and the low-frequency triangular wave generated by the signal generator are superimposed by an adder and then input into the laser driver to control the laser parameters. The electrical signal obtained after the laser irradiates the QTF is transmitted to the lock-in amplifier for 2*f* demodulation. Mixtures of 10% CO_2_ and pure nitrogen (N_2_) are used to prepare different concentrations of target gas for testing.

## 3. Experimental Results and Discussion

**Figure 6:**
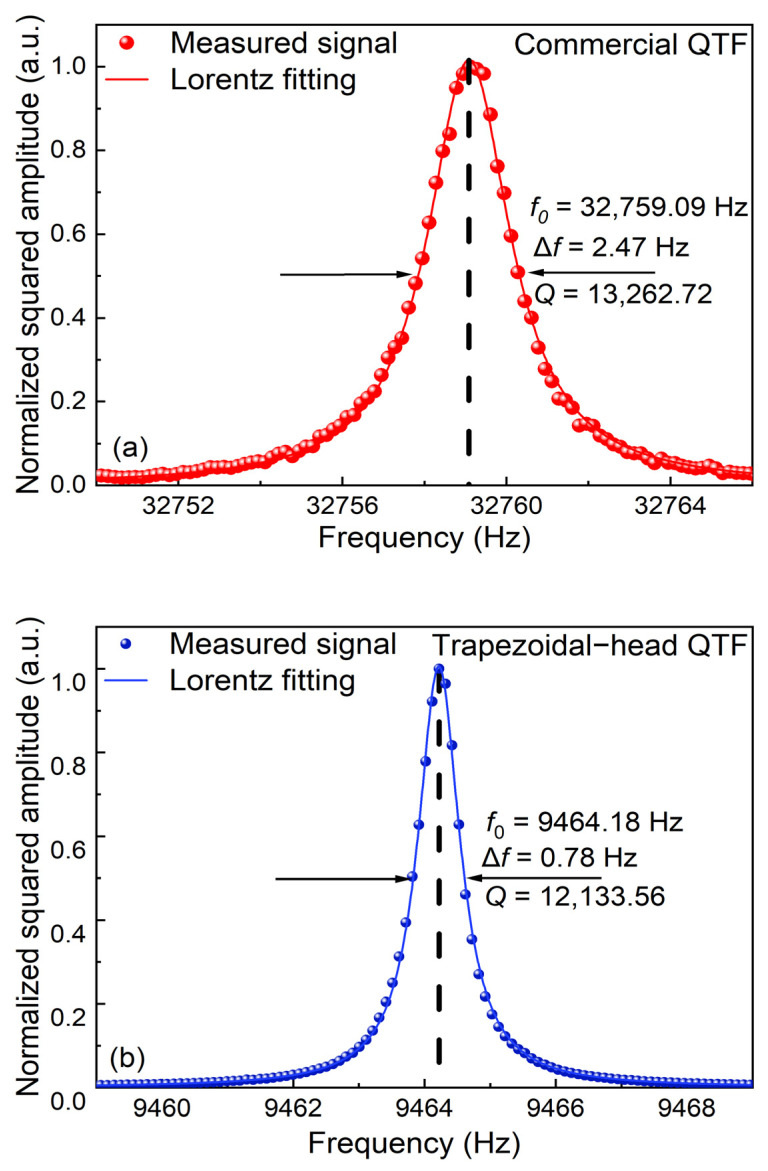
Frequency response characteristic curves of two types of QTF: (**a**) commercial QTF; (**b**) self-designed trapezoidal-head QTF.

Firstly, the response characteristics of the QTF were investigated, and the optical excitation method was employed. After square normalization and Lorentz fitting of the obtained data, the frequency response curve of the commercial QTF is shown in Figure 6a. Its resonant frequency *f*_0_ = 32,769.09 Hz and response bandwidth Δ*f* = 2.47 Hz. According to the equation *Q* = *f*_0_/Δ*f*, *Q* can be calculated as 13,262.72. The frequency response curve of the self-designed trapezoidal-head QTF is shown in Figure 6b. Its resonance frequency, bandwidth, and *Q* are determined as *f*_0_ = 9464.18 Hz, Δ*f* = 0.78 Hz, and *Q* = 12,133.56, respectively. From the relation of energy accumulation time *t*~*Q*/*f*_0_, it can be seen that the energy accumulation time of the self-designed trapezoidal-head QTF is three times longer than the commercial one.

**Figure 7:**
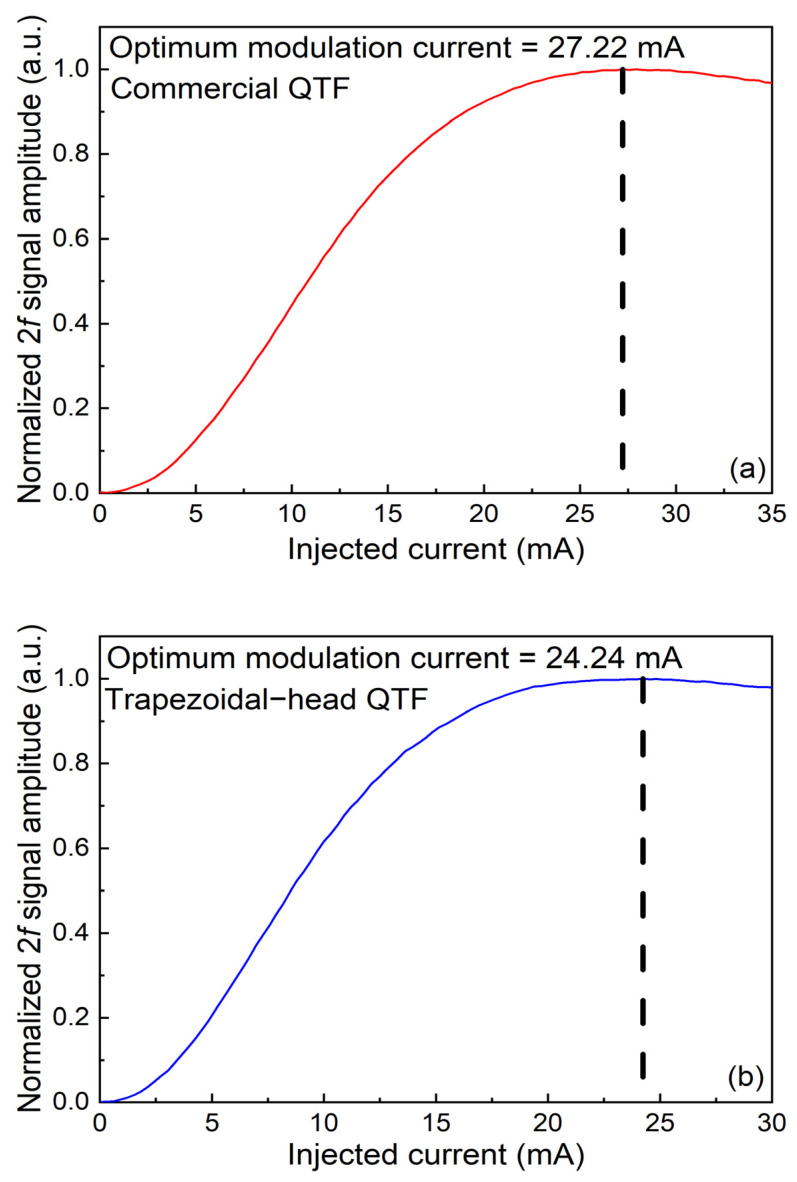
The relationship between current modulation depth and signal amplitude: (**a**) commercial QTF; (**b**) self-designed trapezoidal-head QTF.

The modulation depth plays a crucial role in wavelength modulation spectroscopy. When the laser operates under a constant temperature, the wavelength of the emitted laser is determined by the magnitude of the injected current. Therefore, the magnitude of the modulation depth can also be represented by the injection current. The signal amplitude of the commercial QTF and the self-designed trapezoidal-head QTF varying with the injected current were measured, as shown in Figure 7a for the commercial QTF and Figure 7b for the self-designed trapezoidal-head QTF. It can be seen that as the injected current increases, the signal amplitude gradually increases. After reaching a maximum value, it begins to show a slow downward trend. The optimal modulation current for the commercial QTF is 27.22 mA, and for the self-designed trapezoidal-head QTF, it is 24.24 mA. In the following experiments, these two values of optimal modulation current are adopted.

**Figure 8:**
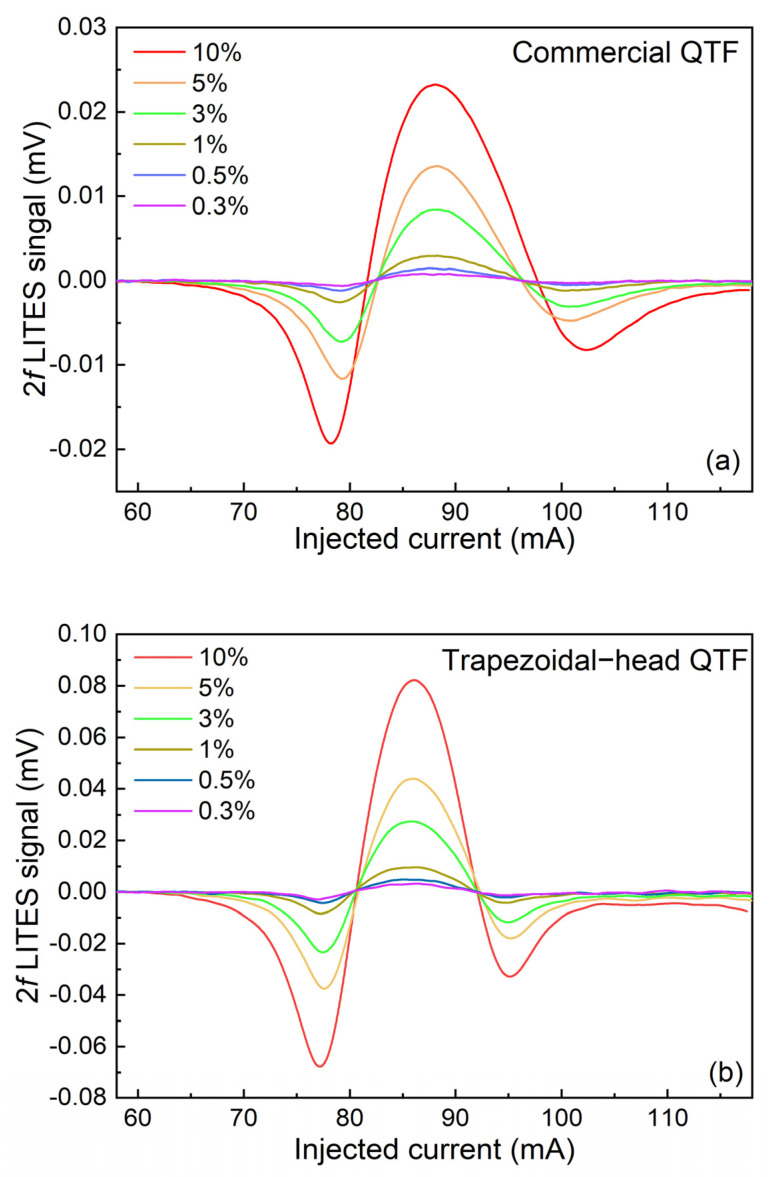
2*f* signals under different CO_2_ concentrations: (**a**) commercial QTF; (**b**) self-designed trapezoidal-head QTF.

The 2*f* measurements are carried out on two types of QTFs using CO_2_ at different concentrations. Figure 8a shows the 2*f* signal of the commercial QTF, and Figure 8b shows the 2*f* signal of the self-designed trapezoidal-head QTF. Two flowmeters are used to control the flow rate of the CO_2_ gas and pure nitrogen (N_2_) to produce different CO_2_ concentrations in the gas cell. From the experimental results, it can be seen that when the CO_2_ concentration is set at 10%, compared with the 2*f* signal of the commercial QTF, the amplitude of the 2*f* signal of the self-designed trapezoidal-head QTF is increased by 3.4 times, which demonstrates the excellent performance of the self-designed trapezoidal-head QTF. Furthermore, from Figure 8, it can be seen that the left and right wings of these 2*f* waveforms are asymmetric, which is caused by residual amplitude modulation.

**Figure 9:**
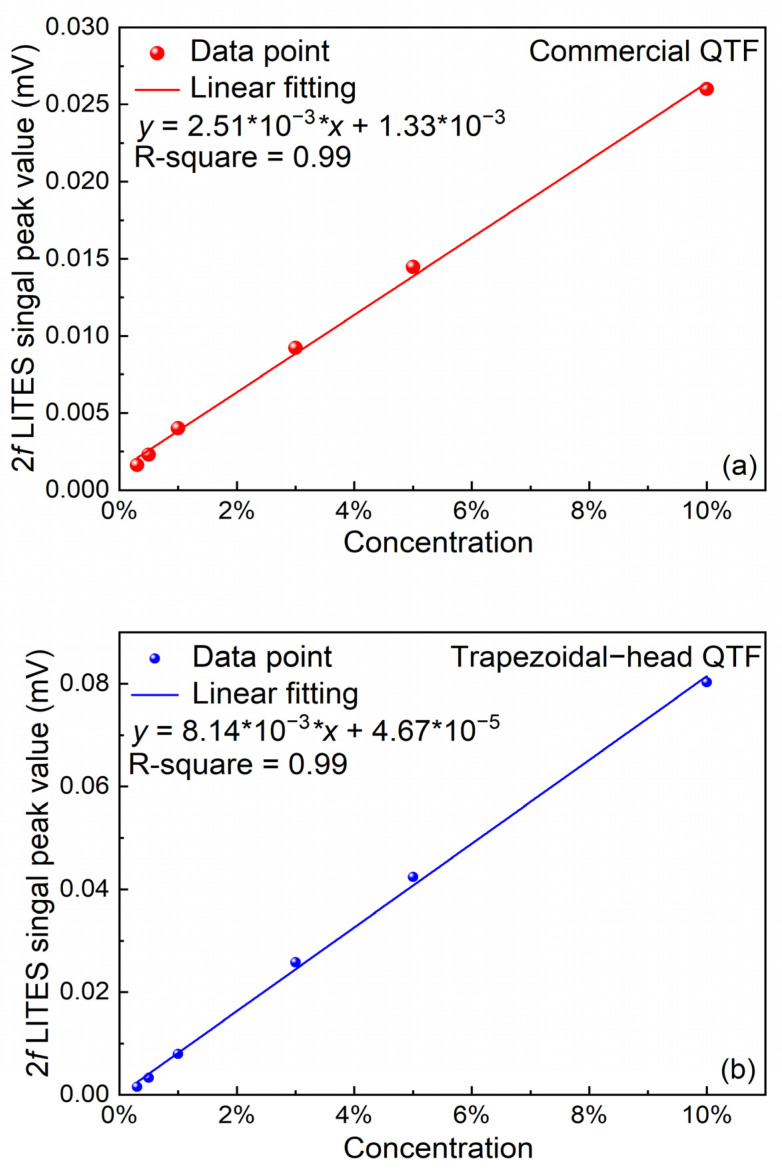
Linear fitting of 2*f* signal amplitudes under different concentrations of CO_2_: (**a**) commercial QTF; (**b**) self-designed trapezoidal-head QTF.

The experimental results show that in the two CO_2_-LITES systems, the concentration of CO_2_ is directly proportional to its own concentration. The amplitudes of the 2*f* signals of the two types of QTFs at different concentrations are linearly fitted. Figure 9a shows the linear fitting of the commercial QTF, and Figure 9b shows the linear fitting of the self-designed trapezoidal-head QTF. Their linear fitting functions are *y* = 2.51 × 10^−3^ × *x* + 1.33 × 10^−3^ and *y* = 8.14 × 10^−3^ × *x* + 4.67 × 10^−5^, respectively. The calculated R-square values of both linear fittings are 0.99, which reflects the excellent linear response of this CO_2_-LITES sensor to the CO_2_ when the two different QTFs are used.

When pure N_2_ is introduced into the gas cell, the noise is measured. For the commercial QTF, the 1σ noise is found to be 32 nV. In this condition, the calculated signal-to-noise ratio (SNR = signal amplitude/noise standard deviation) is 725.97, and the minimum detection limit (MDL) is calculated as 137.75 ppm. For the self-designed trapezoidal-head QTF, the noise and calculated SNR and MDL are 37.6 nV, 2170.2, and 46.08 ppm, respectively.

**Figure 10:**
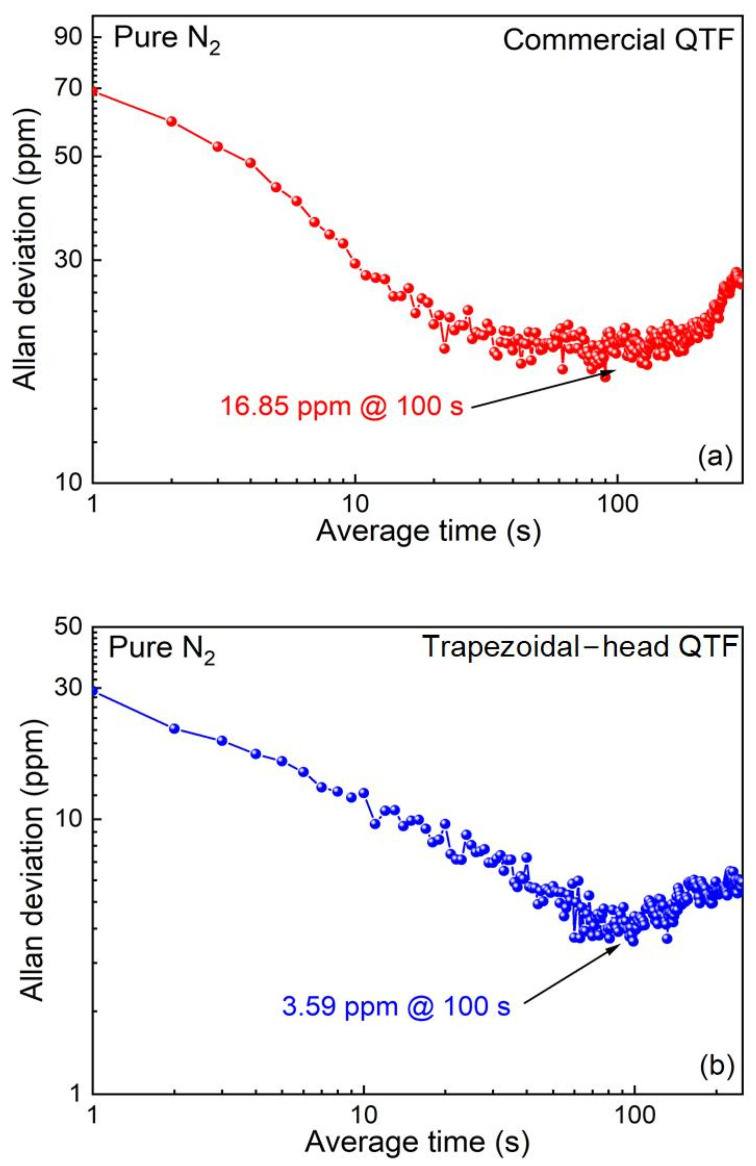
Allan deviation analysis of CO_2_-LITES sensor: (**a**) commercial QTF; (**b**) self-designed trapezoidal-head QTF.

Finally, the Allan variance was used to evaluate the system stability of the CO_2_-LITES sensors with the commercial QTF and the self-designed trapezoidal-head QTF. Pure N_2_ was flushed into the gas cell continuously. Figure 10a shows that when the average time reaches 100 s, the MDL of the LITES sensor based on the commercial QTF improves to 16.85 ppm. Figure 10b shows that when the integration time reaches 100 s, the MDL of the self-designed trapezoidal-head QTF reaches 3.59 ppm. Therefore, after optimization through the Allan variance analysis method, it is found that the minimum detection limit of the self-designed trapezoidal-head QTF is 4.7 times that of the commercial QTF. This investigation demonstrates that both sensors have excellent stability, and the detection ability of the self-designed trapezoidal-head QTF is significantly better than that of the commercial QTF.

## 4. Conclusions

In this paper, a highly sensitive CO_2_-LITES sensor using a 2.004 µm CW-DFB diode laser and a self-designed low-frequency trapezoidal-head QTF is reported for the first time. The QTF has a resonant frequency of 9464.18 Hz, increasing the accumulation time significantly. A comparison between the commercial QTF with a resonant frequency of 32,759.09 Hz and the trapezoidal-head QTF is performed to show the advantages of the self-designed trapezoidal-head QTF. The long-term stability of the system is evaluated using Allan deviation analysis. The experimental results show that when the sensor’s averaging time is increased to 100 s, the MDL for the CO_2_-LITES sensor with the commercial QTF is 16.85 ppm, while for the self-designed trapezoidal-head QTF, the MDL improves to 3.59 ppm. This demonstrates that the self-designed trapezoidal-head QTF significantly enhances the sensor’s detection capability. In the future, the signal can be enhanced by designing new types of QTFs and optimizing their dimensions.

## Data Availability

The data presented in this study are available on request from the corresponding author.
